# Pathology and Pathogenesis of Brain Lesions Produced by *Clostridium perfringens* Type D Epsilon Toxin

**DOI:** 10.3390/ijms23169050

**Published:** 2022-08-12

**Authors:** John W. Finnie, Francisco A. Uzal

**Affiliations:** 1 Office of the Deputy Vice-Chancellor (Research) and Discipline of Anatomy and Pathology, School of Medicine, University of Adelaide, Adelaide, SA 5005, Australia; 2 California Animal Health and Food Safety Laboratory, School of Veterinary Medicine, University of California Davis, San Bernardino, CA 92408, USA

**Keywords:** *Clostridium perfringens* type D, epsilon toxin, neuropathology, pathogenesis

## Abstract

*Clostridium perfringens* type D epsilon toxin (ETX) produces severe, and frequently fatal, neurologic disease in ruminant livestock. The disorder is of worldwide distribution and, although vaccination has reduced its prevalence, ETX still causes substantial economic loss in livestock enterprises. The toxin is produced in the intestine as a relatively inactive prototoxin, which is subsequently fully enzymatically activated to ETX. When changed conditions in the intestinal milieu, particularly starch overload, favor rapid proliferation of this clostridial bacterium, large amounts of ETX can be elaborated. When sufficient toxin is absorbed from the intestine into the systemic circulation and reaches the brain, two neurologic syndromes can develop from this enterotoxemia. If the brain is exposed to large amounts of ETX, the lesions are fundamentally vasculocentric. The neurotoxin binds to microvascular endothelial receptors and other brain cells, the resulting damage causing increased vascular permeability and extravasation of plasma protein and abundant fluid into the brain parenchyma. While plasma protein, particularly albumin, pools largely perivascularly, the vasogenic edema becomes widely distributed in the brain, leading to a marked rise in intracranial pressure, coma, sometimes cerebellar herniation, and, eventually, often death. When smaller quantities of ETX are absorbed into the bloodstream, or livestock are partially immune, a more protracted clinical course ensues. The resulting brain injury is characterized by bilaterally symmetrical necrotic foci in certain selectively vulnerable neuroanatomic sites, termed focal symmetrical encephalomalacia. ETX has also been internationally listed as a potential bioterrorism agent. Although there are no confirmed human cases of ETX intoxication, the relatively wide species susceptibility to this toxin and its high toxicity mean it is likely that human populations would also be vulnerable to its neurotoxic actions. While the pathogenesis of ETX toxicity in the brain is incompletely understood, the putative mechanisms involved in neural lesion development are discussed.

## 1. Introduction

There are seven toxinotypes of the anaerobic, spore-forming bacillus, *Clostridium perfringens*, classified as A, B, C, D, E, F and G based on their elaboration of one or more of six principal exotoxins, namely, alpha (CPA), beta (CPB), epsilon (ETX), iota (ITX), enterotoxin (CPE) and necrotic enteritis B-like (Net-B) (Table 1) [1]. Type D strains produce two of these principal toxins (CPA and ETX), but ETX is considered the main virulence factor of this toxinotype, as demonstrated by the fulfillment of the molecular Koch postulates in several experimental animal models [2,3].

*Clostridium perfringens* type D enterotoxemia is one of the most common clostridial diseases of sheep and goats, and is of worldwide distribution [4,5,6]. Since the disease is often fatal, it can cause significant economic loss to livestock enterprises, particularly if animals are unvaccinated. Vaccination is also a significant expense for livestock producers. The disease occurs less commonly in cattle [7,8,9], and very infrequently in deer and perhaps domesticated camels, and horses [10,11]. However, although type D enterotoxemia is an infectious disease that can occur in the form of small outbreaks, it is not a contagious disease. ETX has also been placed on several international bioterrorism lists as it is a potential, albeit unproven, agent of zoonotic disease. Moreover, there is some serologic evidence that ETX may be implicated in the development of multiple sclerosis [12], although the neuropathology of this chronic and progressive human demyelinating disease is very different from the acute or subacute disease produced by ETX in ruminant livestock and laboratory rodents.

**Figure 1:**
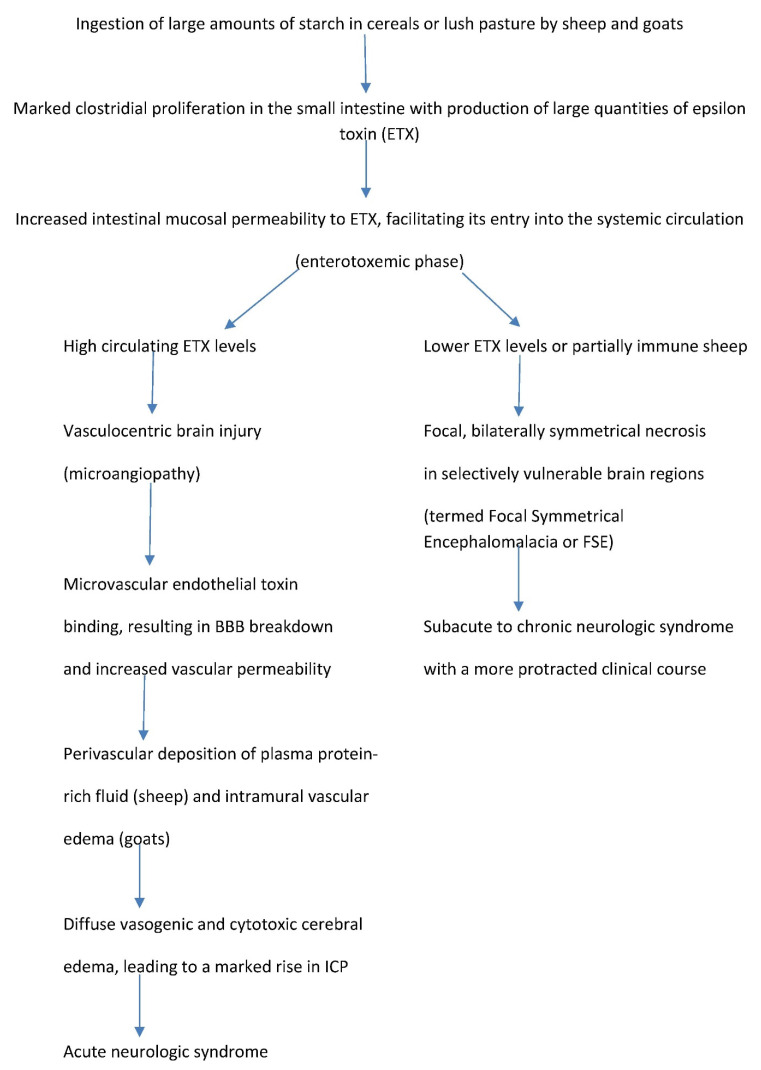
Pathogenesis of *Clostridium perfringens* type D epsilon toxin neurotoxicity in ruminant livestock.

The temporal sequence of development of the disease produced in ruminant livestock by *Clostridium perfringens* type D epsilon toxin commences with changes in the intestinal milieu that favor massive proliferation of this resident bacterium, resulting in the production of large quantities of ETX (Figure 1). This toxin then facilitates its own absorption into the systemic circulation by increasing intestinal permeability, thereby giving rise to a purely enterotoxemic phase [13]. The circulating toxin binds to the microvascular endothelium of several tissues, including lung, heart, kidney and eye, but is particularly injurious to blood vessels in the brain [14]. When the cerebral vasculature is exposed to large amounts of ETX, the resulting increased vascular permeability permits marked extravasation of plasma protein and fluids into the subintimal wall of affected blood vessels and the perivascular space. The ensuing severe, generalized, parenchymal edema is initially vasogenic, then a mix of vasogenic and cytotoxic. This edema produces a marked increase in intracranial pressure and an acute, and often fatal, neurologic syndrome [15]. However, when the blood–brain barrier (BBB) is exposed to smaller quantities of ETX, or the intoxicated animal is partially immune, a more protracted clinical course sometimes follows, with the formation of bilaterally symmetrical necrotic lesions in neuroanatomic regions of predilection. This subacute or chronic intoxication is termed focal symmetrical encephalomalacia (FSE) [6,16,17,18,19,20]. The sequence of pathogenic events leading to the different clinical manifestations of ETX neurotoxicity in sheep and goats is shown in Figure 1.

## 2. Epsilon Toxin

Clostridial diseases can be divided into three categories, based on toxin activity and tissues affected. These are neurotoxic, histotoxic and enteric diseases. Neurotoxic clostridia, such as *Clostridium tetani* and *Clostridium botulinum*, affect neuromuscular function, without producing any observable gross or microscopic tissue damage. By contrast, histotoxic clostridia, for example, *Clostridium chauvoei*, *Clostridium septicum*, *Clostridium novyi* and *Paeniclostridium sordellii*, produce localized lesions in tissues such as muscle and liver, and may subsequently become toxemic. The third group, enteric diseases, comprises *Clostridium perfringens*, *Clostridium difficile*, *Clostridium colinum* and others. In sheep and goats, *Clostridium perfringens* is the most significant enteric clostridial pathogen. Each type of this microorganism (A through G) produces immunologically distinct major exotoxins, some of which cause inflammatory lesions in the gastrointestinal tract and/or enterotoxemia. A range of other toxins, some of which enhance virulence, but are not used in the classification of *Clostridium perfringens*, is also recognized. The pattern of toxin production varies with each *Clostridium perfringens* type and determines the ensuing clinical syndrome, with sustained levels of clostridial exotoxin usually being required for the development of systemic clinical signs. Clostridial infections invariably need favorable predisposing conditions which, in the case of *Clostridium perfringens* type D, is an alteration to the intestinal microbiota caused mostly by an abrupt change to the feed type, namely, large amounts of grain or succulent pasture [16,21,22,23].

*Clostridium perfringens* type D secretes a relatively inactive, ~33 kDa polypeptide, termed epsilon prototoxin (pETX). This prototoxin is proteolytically activated by intestinal enzymes, or other proteases such as lambda toxin produced by some strains of *C. perfringens*, to the active ~29 kDa ETX, by removing C-terminal amino acids [24,25,26]. ETX is produced during log-phase growth, but the regulation of its production is incompletely understood [27].

ETX is a member of the aerolysin family of pore-forming toxins, which oligomerize at the cell surface, form pores of varying size in the membrane and eventually cause cell lysis. One domain of ETX interacts with receptors on the host cell, while another domain possesses an amphipathic loop that inserts into membranes during pore formation. Once bound to a receptor, ETX uses lipid rafts and caveolins to oligomerize into a heptameric pre-pore on the host cell surface, then extends a β-hairpin loop into the membrane lipid bilayer to form an active pore. This pore formation results in a rapid decrease in cytoplasmic K^+^ levels, with a consequent influx of Na^+^ and Cl^−^ into the cell, leading to cell necrosis [2,28].

## 3. Intestinal Bacterial Proliferation and Toxin Production

A small number of ruminants harbor *Clostridium perfringens* type D in their small intestine, but bacterial numbers are generally small, in part due to peristalsis. Clinical disease does not occur unless the microbial balance in the gut milieu is disrupted. The small amounts of ETX normally present in the intestinal lumen appear to be relatively harmless [16,22,23]. There is, nevertheless, often sufficient circulating antibodies, abetted by vaccination, in lambs, goat kids and older sheep and goats to make experimental reproduction of this disease difficult, frequently necessitating the use of colostrum-deprived lambs or goat kids. Laboratory rodents are also frequently used for this purpose.

When sheep and goats, and more particularly lambs, graze abundant lush pasture or young cereal crops, or after heavy grain feeding in feedlots, large quantities of undigested starch pass into the small intestine. Since it takes days or weeks for the rumen microflora to adapt to this sudden overload of ingested carbohydrates, the starch overload, which has given the disease the colloquial designation “overeating disease”, provides an excellent substrate for these saccharolytic clostridial bacteria, and they proliferate rapidly. Large amounts of ETX are produced. However, while the presence of excess starch in the small intestine encourages clostridial proliferation, there is experimental evidence that, in fact, it is a lack of glucose in this microenvironment, due to a failure to digest the starch overload, which appears to stimulate ETX production [16,22,23].

## 4. The Enterotoxemic Phase

Type D enterotoxemia in sheep, and likely cattle, is a pure toxemia, with no bacterial invasion of tissues. Large amounts of ETX in the intestinal lumen appear to increase mucosal permeability, thus facilitating toxin absorption into the bloodstream (Goldstein et al., 2009 [13]). When a high luminal concentration of ETX is sustained, sufficient toxin enters the circulation to produce neurologic, respiratory, and perhaps cardiac dysfunction. By contrast, the disease in goats can be either a pure enterotoxemia, a localized enteric condition, or a combination of both. The enteric lesions in goats are probably due to a localized toxic action of ETX and present as subacute or chronic forms of the disease [16,22,23].

The circulating toxin is distributed to a range of target tissues, including brain, lungs, liver, heart, kidneys and eye, where it binds to a receptor. This binding can be experimentally, albeit transiently, prevented via competitive inhibition by prior injection of pETX [29,30,31,32]. ETX binding has been reported in a variety of tissues, including renal tubules [30,33,34], myelin [35,36,37,38] and brain endothelial cells [34,35,39]. ETX binding to most cells is believed to be mediated by expression of the myelin and lymphocyte protein (MAL), which is the current proposed ETX receptor [40]. MAL is a transmembrane, highly hydrophobic proteolipid with two extracellular loops. It has been recently proposed that MAL expression is necessary, and sufficient, for ETX binding and cytotoxicity. However, expression of MAL in tissue of natural hosts of *Clostridium perfringens* type D enterotoxemia has not yet been determined.

*Clostridium perfringens* type D enterotoxemia of sheep spans a disease continuum, ranging from a peracute clinical course with sudden death at the severe end of the spectrum to a subacute or chronic intoxication with a more prolonged course. When large amounts of ETX are absorbed from the intestine, the disease can be rapidly fatal [41].

## 5. The Acute Neurologic Syndrome: Vasculocentric Brain Injury with Resulting Generalized Cerebral Edema

### 5.1. Neurologic Signs

Acute ETX neurotoxicity mainly affects young, rapidly growing lambs and goat kids, but sheep and goats of all ages may succumb. It was traditionally believed that newborn lambs and kid goats are not susceptible to type D enterotoxemia, probably because of the low level of trypsin activity in the intestine due to the trypsin inhibitory action of colostrum. However, recent evidence indicates that rare cases may occur in newborn lambs and goat kids colonized by lambda toxin-positive strains of *Clostridium perfringens* type D [18]. In these cases, it was assumed that lambda toxin was responsible for pETX activation in the absence of trypsin. Morbidity can be up to 10%, but lethality approaches 100%. The illness in young lambs, especially unvaccinated animals without circulating antitoxin, is very brief, often less than 2 h, but usually not exceeding 12 h. Many lambs are found dead without premonitory signs or die after a few minutes of violent convulsive activity. Cases surviving for a few hours may show diarrhea, tenesmus, tachypnea, ptyalism, staggering, hyperesthesia, ophistotonus, convulsions and coma. Clinical signs are similar in goat kids and calves [16,22,23].

At autopsy, lamb carcasses are usually well-nourished. There is frequently excess, straw-colored pericardial fluid, which clots after being exposed to air, and forms fibrin strands. Pulmonary congestion and edema, as well as hemorrhage beneath the endocardium and epicardium of the left ventricle and other serosal membranes, are common macroscopic findings. Kidneys may be soft, friable and congested with type D enterotoxemia, sometimes referred to as “pulpy kidney disease”. However, since this gross appearance probably reflects accelerated autolysis, its diagnostic importance should not be overestimated, especially if the autopsy is delayed. There is usually scant evidence of enteritis, but segments of small intestine are sometimes distended with gas and the mucosa is hyperemic [16,22,23,42,43]. Gross findings are similar in goats and cattle.

Hyperglycemia and glycosuria, the result of rapid mobilization of hepatic glycogen, are pertinent clinical chemistry diagnostic aids. However, they are not consistently present, especially if urine collection is delayed post-mortem, or etiologically specific, being found in the terminal stages of other, particularly hepatic, ruminant disorders. A diagnosis of type D enterotoxemia is also abetted by smears of the intestinal mucosa containing large numbers of proliferating, Gram-positive, rod-shaped bacteria, which then need to be positively identified as *C. perfringens* by anaerobic bacteriologic culture [44].

The presence of a large quantity of ETX in the intestinal contents is confirmatory of ETX poisoning, although small amounts of toxin and numbers of *C. perfringens* type D can sometimes be found in clinically normal sheep. These small quantities of ETX are, nevertheless, below the detection limit of most routine laboratory diagnostic tests used to detect ETX. A few techniques have been used to detect ETX in the intestinal contents of sheep and goats, including polyclonal capture enzyme-linked immunosorbent assay (ELISA), monoclonal capture ELISA, a mouse neutralization test, and counterimmunoelectrophoresis. However, the results of these tests have sometimes been inconsistent [45,46,47] and, accordingly, they should be used in concert with clinicopathologic assessments. 

### 5.2. Neuropathology

**Figure 2:**
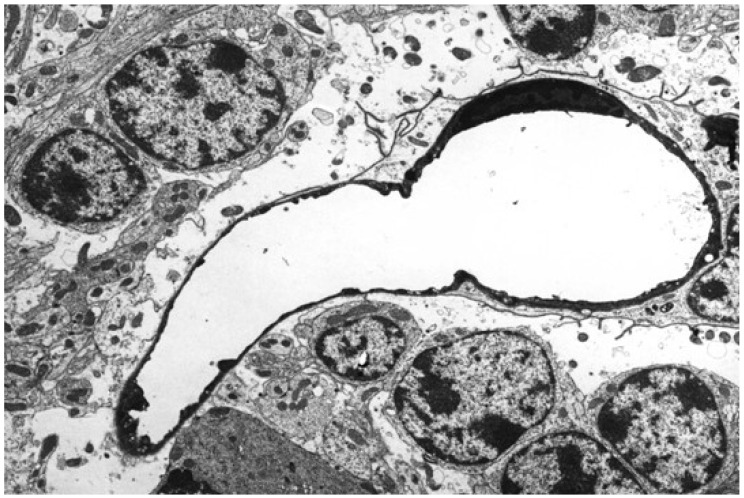
Transmission electron micrograph of a capillary in an acutely ETX-intoxicated ovine brain showing marked endothelial attenuation and electron density (coagulation necrosis) with nuclear pyknosis. Uranyl acetate and lead citrate. Reproduced from Uzal et al., 2016.

In peracutely, acutely and subacutely intoxicated sheep, and to a lesser degree, goats, the brain microvascular endothelium forming the BBB appears to be the principal target of ETX neurotoxicity [14,15,22,23,28,37,48,49,50]. Ultrastructural examination of ETX-injured cerebral microvessels reveals endothelial swelling initially, with loss of cytoplasmic organelles and blebbing of the luminal surface, which progresses to an attenuated, electron-dense cytoplasm, corresponding to coagulation necrosis and nuclear pyknosis (Figure 2) [47,51,52,53,54,55,56].

**Figure 3:**
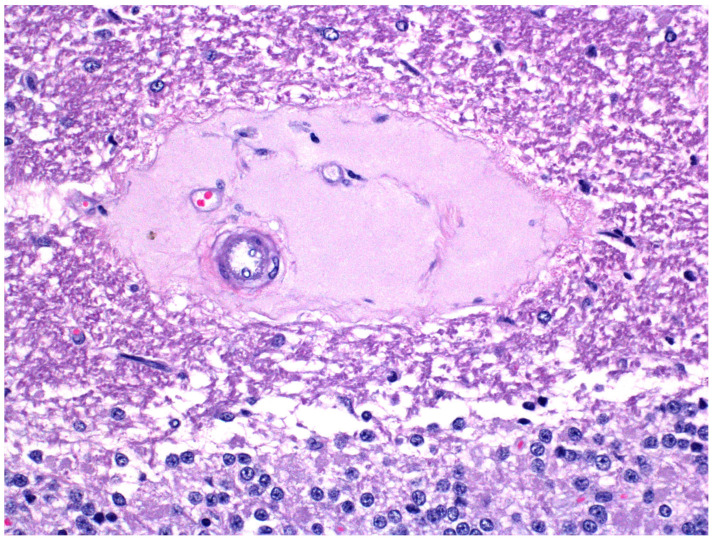
Microangiopathy with perivascular deposition of extravasated protein-rich fluid in acute ovine ETX intoxication. H&E.

These fine structural changes are also found in ETX-exposed pulmonary, hepatic, renal and retinal microvessels [43,57,58,59,60]. Brain microvessel endothelial damage results in the accumulation of perivascular and intramural lakes of albumin-rich, plasma protein-derived fluid (Figure 3), which is typical of type D disease, and a diffusely distributed parenchymal vasogenic edema [15,61,62]. This edema is more commonly found in white than gray matter, particularly in the cerebral cortex, corpus striatum, thalamus, midbrain, medulla and cerebellum. The sparse cellularity and large extracellular spaces in white matter permit greater accumulation and spread of this vasogenic edema fluid in the brain [49,63].

In ETX-intoxicated goats, microvascular intramural accumulation of proteinaceous edema fluid is more often found in the brain than the perivascular lakes that are a common histologic feature in ovine cases. This is posited to be due to the lower doses of toxin absorbed from the intestine in goats and, consequently, less severe microvascular injury and BBB breakdown [64,65].

It has recently been confirmed [43] that only ETX-producing strains of *Clostridium perfringens* cause acute cardiopulmonary lesions, of varying severity, in sheep. Death can occur very rapidly when these lesions are severe and, in addition to brain damage, cardiac dysfunction caused by ETX is a potential cause of mortality. Hydropericardium (with a high fibrin content) and pulmonary edema are the most common cardiopulmonary lesions, followed by hydrothorax, the latter being highly correlated with interstitial pulmonary edema. Endocardial and myocardial hemorrhage are also frequently found. Cardiomyocyte necrosis is common, especially in the region of the atrioventricular node, and this site predilection could lead to cardiac dysfunction and be responsible for marked clinical deterioration and even death [43].

### 5.3. Pathogenesis

The primacy of microvascular damage in the pathogenesis of acute EXT neurotoxicity is supported by the fact that this toxin produces a rapid and dose-dependent cytotoxic effect on cerebral microvascular endothelial cells in vitro [66,67]. Furthermore, brain and retinal vasogenic edema in rats given ETX is secondary to loss of capillary endothelial barrier antigen (EBA), EBA being a marker of an intact BBB and blood–retinal barrier in this species [58,59,60,68].

However, although these vasculocentric histologic changes are found in acutely to subacutely ETX intoxicated ruminants, and support a diagnosis of type D enterotoxemia, it is not known why there is an inconsistency, in both frequency and neuroanatomic distribution, of vascular lesions between individual cases. It is possible that only a subset of cerebral blood vessels possesses endothelial receptors for ETX or, given that ETX neurotoxicity is expressed in a dose- and time-dependent manner, these factors could determine which microvessels in selected brain areas are exposed to sufficient toxin to sustain endothelial damage. In experimentally ETX-intoxicated laboratory rodents, the toxin has been shown to cause either selective or more widespread neural injury, depending on the dose of circulating ETX and the time interval between toxin administration and death. In general, higher doses of ETX tend to expand the distribution of brain lesions, although still favoring certain vulnerable areas, whereas lesions in sub-acutely intoxicated animals exposed to lower doses of ETX are smaller, tend to be well-circumscribed and are confined to fewer susceptible brain regions. Moreover, as the time from toxin administration to death increases, the distribution of brain lesions tends to become more widely distributed [14]. Another possible reason for the lack of uniformity in microvascular damage produced in the brain by ETX is the recognition that cerebral endothelial cells constitute a heterogeneous population, this variability being attributed to the functional diversity between different brain regions and among capillaries and venules, the latter evident even at the single cell level within individual microvessels [69].

In acute ETX intoxication, the brain edema due to breakdown of the BBB is initially vasogenic. This microvascular incompetence permits abundant extravasation of plasma proteins and fluid into the extracellular space (ECS). The protein-rich fluid principally affects the white matter, extending from the gray matter along adjacent white matter tracts. White matter edema is facilitated by the freedom with which fluid can move through parallel fiber bundles, and this edema progression can result in tissue necrosis. However, excessive water accumulation and dissemination eventually become a mix of vasogenic (interstitial) and cytotoxic (cellular) edema, the latter characterized by intracellular accumulation of water, mainly in astrocytes, and then in neurons [70].

Cerebral edema is a severe, deleterious and frequently fatal result of ETX-induced microvascular injury. It is defined as an increase in brain volume due to an elevation of tissue water content and results from the passage of free water from the vascular compartment to an intracellular or extracellular site, or both. This definition does not differentiate between either location, although it has been argued that cytotoxic edema is not a true edema as it is intracellular rather than interstitial [70]. Brain edema is considered to cause neurologic dysfunction only when it is sufficiently severe to cause ischemia or shift, distortion and herniation of the brain, or when the edema fluid transports deleterious factors that result in localized neural injury [70].

**Figure 4:**
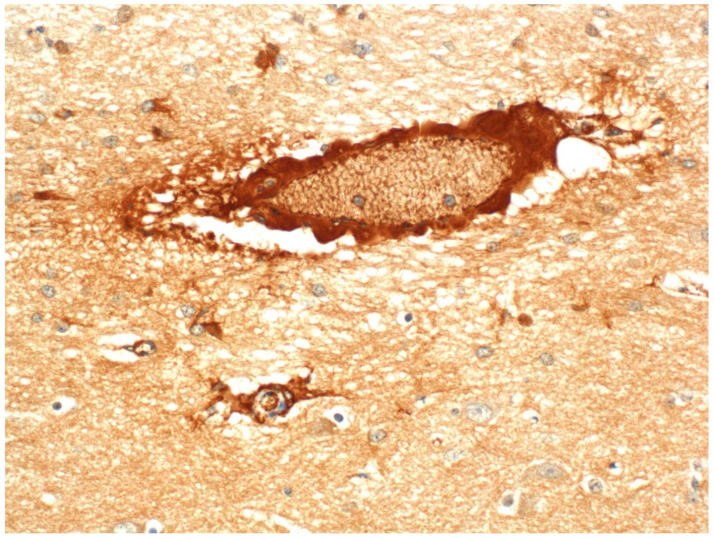
Perivascular accumulation of extravasated plasma albumin as a surrogate marker of increased vascular permeability in acute ovine ETX intoxication. Albumin immunohistochemistry.

In acute ETX intoxication, there is widespread extravasation of plasma albumin (Figure 4), which has been used as a surrogate immunohistochemical marker of increased vascular permeability in both diagnostic and experimental studies [15,55,56,58,59,65], albumin being the smallest (66 kDa), but most abundant, of the plasma proteins. This plasma-rich extravasation also correlates with increased, and widely distributed, aquaporin-4 (AQP-4) expression in astrocytes, AQP-4 being the major water channel protein in the brain. In addition to participating in the development of brain edema, AQP-4 upregulation appears to play a role in reducing this edema by moving water from the ECS into astrocytes, thus reducing osmotic stress on surrounding neurons [56].

## 6. The Protracted Neurologic Syndrome: Focal, Bilaterally Symmetrical, Regionally Selective, Neuroparenchymal Necrosis

### 6.1. Neurologic Signs

Sheep that develop FSE lesions usually survive for 5 to 7 days, and sometimes up to 14 days, and this more chronic form of ETX intoxication is characterized by neurologic signs including blindness, aimless wandering, ataxia, bruxism, head pressing, nystagmus, posterior paresis, lateral recumbency, ophistotonus and paddling convulsions [16,20,71].

Lambs and older sheep with subacute or chronic ETX intoxication may show herniation of the cerebellar vermis through the foramen magnum (“cerebellar coning”) which, although highly suggestive of type D enterotoxemia, can also occur with other disorders causing raised intracranial pressure [16].

In goats, both kids and adult animals may be affected, and acute, subacute and chronic forms of the disease are recognized [72,73,74]. The acute disease resembles that occurring in lambs and usually affects young, unvaccinated animals. The subacute form in older goats is characterized by diarrhea, which may be hemorrhagic, and severe abdominal discomfort. There may be neurologic or respiratory signs. The chronic form occurs mainly in vaccinated adult animals and may persist for a few days or weeks. These goats sometimes die but can recover. Clinically, there is profuse, watery and/or hemorrhagic diarrhea and, at autopsy, there is severe colitis, which sometimes extends into the small intestine as an enterocolitis. The colonic lumen is often distended, the contents containing mucus, fibrin and often frank blood; the mucosa is necrotic and sometimes covered by a fibrinous pseudomembrane. It has been postulated that enteric lesions are more severe in goats than sheep because the intestinal absorption of ETX into the bloodstream is slower in the former species, resulting in more localized enteric damage [74,75]. This, however, has not been proved and remains speculative.

### 6.2. Neuropathology

**Figure 5:**
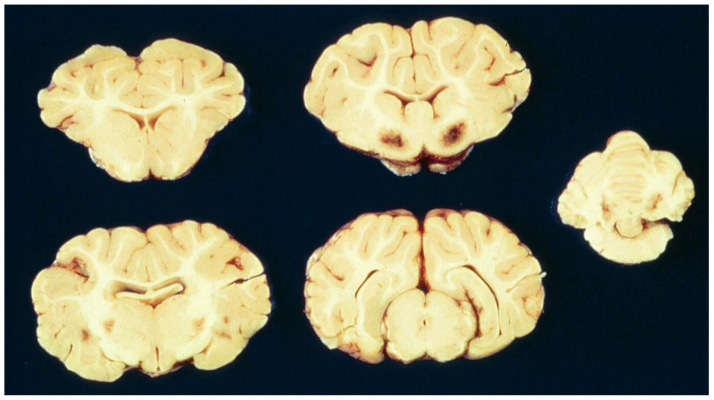
Focal symmetrical encephalomalacia (FSE) in a sheep showing bilaterally symmetrical necrotic foci in the corpus striatum, thalamus and cerebellar peduncles. Photo courtesy of B. Hartley.

In ETX-exposed sheep following a more protracted clinical course, bilaterally symmetrical foci of necrosis are sometimes found in certain brain regions of predilection, including the basal ganglia, thalamus, internal capsule, midbrain, medulla oblongata, and cerebellar peduncles (Figure 5), this neuropathologic entity being termed FSE [20,76,77,78]. While these focal, sometimes hemorrhagic, softenings (malacia) have a similar morphologic expression to those found in a few other ruminant neurologic diseases, the different neuroanatomic patterns of lesion distribution in the respective conditions can be diagnostically very useful [5,79].

### 6.3. Pathogenesis

While FSE is pathognomonic for the more chronic manifestation of type D disease in sheep, the reason for the selective vulnerability of certain brain regions remains elusive. It could reflect the higher susceptibility to ETX of the neural parenchyma, or vasculature, in these areas. After gaining access to the brain by damaging the microvasculature, it is possible that ETX could have a differential cytotoxic action on specific neuroanatomic sites [80]. However, no ETX-induced hippocampal neuronal cytotoxicity was found in vitro [81], although these were neuronal cultures from 19-day-old fetal rat brains, which may not replicate any neuronal ETX toxicity in vivo. The sizable necrotic lesions in FSE also suggest a localized failure of perfusion, but obstruction of a large area of the microvascular bed, or occlusion of perforating end-arteries, would be required, and neither of these vascular events have been demonstrated to date.

**Figure 6:**
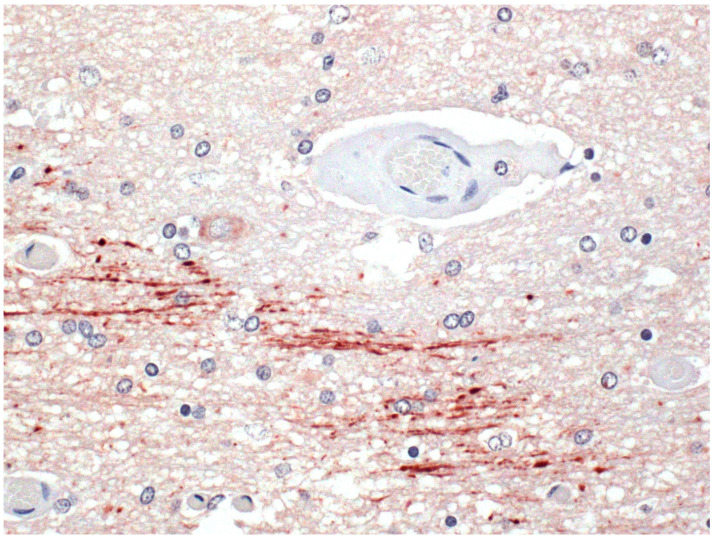
Focal symmetrical encephalomalacia. A focal necrotic area in the internal capsule showing abundant amyloid precursor protein (APP)-immunopositive inured axons in an area surrounding a blood vessel with perivascular edema. APP immunohistochemistry.

Studies of ETX pathogenesis in laboratory rodents have shown that this toxin causes excessive release of glutamate from pre-synaptic, glutamate-rich sites in the hippocampus, leading to post-synaptic dendritic injury and pyramidal cell death. The toxin also binds to the soma and dendrites of glutamatergic cerebellar granule cells and damages dopaminergic areas of the brain [36,82,83,84]. However, whether these findings are translatable to naturally occurring ruminant cases remains to be determined. Similarly, ETX has been shown to bind to, and kill, oligodendrocytes in a dose- and time-dependent manner in brain cultures [38] and bind to myelin [35], but whether these toxic actions contribute to the development of demyelinating lesions in human brain diseases, such as multiple sclerosis, is yet unknown. In areas of necrosis there is overexpression of amyloid precursor protein (APP) highlighting injured axons (Figure 6).

The development of focal necrotic lesions in only some brain regions in FSE conforms to the concept of selective vulnerability [85]. In most neurologic diseases, including ETX intoxication, an insult is delivered to the entire brain, but damage is confined to only a subset of neurons in certain regions, and the resulting lesion distribution tends to be characteristic of that disease. These lesions are largely a consequence of the fact that the brain is the most inhomogeneous of the organs in the body and, only in exceptional circumstances, does a disease uniformly affect all regions of the brain [85].

While a cogent explanation for selective vulnerability is lacking for most neuropathologic entities, these lesions can, nevertheless, be diagnostically useful and may provide some insight into pathogenetic mechanisms. In FSE, the malacia (necrosis) denotes a grossly observable softening of the brain, but the malacic process is not pathognomonic for this clostridial disease, being the final common pathway of a variety of toxic, vitamin and nutritional deficiency, and ischemic insults to the central nervous system in domestic animals. However, there is valuable diagnostic specificity in the neuroanatomic pattern of distribution of FSE lesions [5].

In addition to FSE, there are a few examples in domestic animals of well-circumscribed, bilaterally symmetrical, selective necrosis in specific gray matter areas of the brain and spinal cord, for example, in the Australian cattle dog, Alaskan Husky, Yorkshire Terrier and Angus and Simmental cattle [79]. A similar focal, bilaterally distributed, lesion pattern is also found in nigropallidal encephalomalacia of horses poisoned by yellow star thistle (*Centaurea solstitialis*) and pigs with selenium toxicoses [5]. Sheep in West Africa, goats in California and Ayrshire calves in the United Kingdom may show similar lesions, although their etiology has not been determined [5].

While reasons for selective vulnerability in most neurologic disorders is not understood, there are a few neuropathologic examples in humans where the selective vulnerability of a particular brain region is striking and correlated with a known pathogenic factor. In carbon monoxide (CO) poisoning, the well-defined necrosis in the globus pallidus and substantia nigra is due to the high affinity of the CO molecule for these iron-rich areas of the brain. Moreover, in transient global ischemia, the copious release and accumulation of the neurotransmitter, glutamate, into the brain extracellular fluid overstimulates N-methyl-D-aspartate (NMDA) receptors in certain hippocampal neurons, leading to excitotoxic death [70]. In order to explain the selective vulnerability of the human brain to hypoxia, two hypotheses were advanced. The vascular theory of Spielmeyer suggested that the anatomical features of some blood vessels in certain brain regions were important underlying factors, while the concept of “pathoclisis” proposed by Vogt argued that physicochemical properties explained localized vulnerabilities to hypoxic injury. However, neither theory fully explained all regional susceptibilities [70]. It is now believed that, in general, a satisfactory explanation for selective vulnerability will probably largely depend on a better understanding of the molecular constituents that are unique to certain brain regions and their interaction with microenvironmental perturbations impinging on neurons in these areas. These molecular characteristics have not yet been determined for FSE-affected brain regions [70].

**Figure 7:**
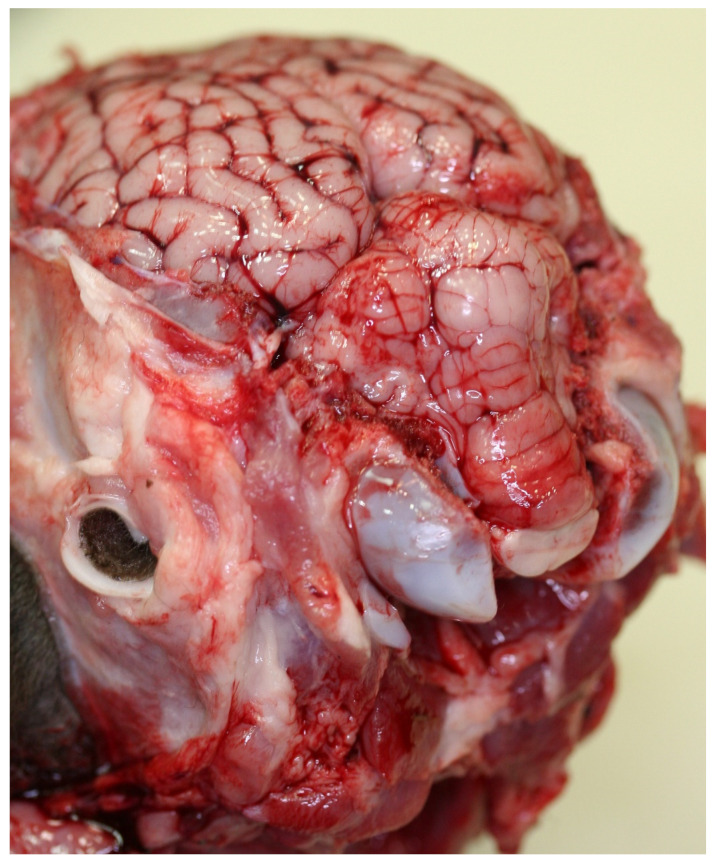
Herniation of the cerebellar vermis through the foramen magnum (“cerebellar coning”) in an ETX-intoxicated sheep. Photo courtesy of B. Barr.

In ruminant ETX intoxication cases, there is sometimes displacement of the cerebellar tonsils through the foramen magnum, termed cerebellar coning (Figure 7). While this herniation may cause apnea, it is probable—in humans, at least—that there is already loss of consciousness at this stage of the clinical course. A marked rise in intracranial pressure (ICP) is a potentially life-threatening event, and although a reduction in the volume of intracranial blood and cerebrospinal fluid can, for a time, prevent or ameliorate this pressure increase (according to the Monro–Kellie doctrine), this compensatory capacity will eventually be exhausted. When this occurs, ICP often inexorably rises, finally reaching levels approaching arterial blood pressure, resulting in decreased cerebral perfusion and, often, cerebellar herniation [86].

In terms of disease prevention measures, vaccination with an alum-adsorbed, ETX toxoid produces solid immunity in sheep and goats and has been effective in reducing the prevalence of this disease. In sheep, two doses of vaccine, administered 4–6 weeks apart, are generally protective for about 12 months; an annual booster is recommended. Lambs from vaccinated ewes are usually given their first immunization at 4–6 weeks of age. Since the immunity produced in vaccinated goats is lower, and of shorter duration, than that of sheep, a booster is recommended every 3–4 months in this species. Pregnant small ruminants are usually vaccinated 2–4 weeks prior to parturition in order to provide colostral immunity to lambs and goat kids [16]. Epsilon antitoxin can potentially be given to sheep and goats when an outbreak commences and circulating antitoxin has been reported to be protective for 20–30 days, but this therapy is not feasible under most field conditions. Hyperimmune serum has also been proposed as a short-term prophylactic measure but, again, is rarely used [42]. However, in the case of human ETX intoxication, both antitoxin and hyperimmune serum would probably be considered as therapeutic interventions.

It is also advisable, especially with unvaccinated animals, to prevent unrestricted access of lambs to large amounts of starch-rich feed, a major predisposing nutritional factor to *C. perfringens* overgrowth, with excessive ETX production, in the intestinal milieu [16].

## 7. Conclusions

In ruminant livestock acutely intoxicated with ETX, microvascular endothelial (BBB) damage appears to be the fundamental brain lesion, resulting in diffuse parenchymal edema, and a resulting marked and rapid rise in ICP. The finding at autopsy of severe pulmonary edema, with hydrothorax and ascites, abundant pericardial fluid which forms fibrin clots when the pericardial sac is opened, and subendocardial hemorrhages are diagnostically useful for a diagnosis of acute ovine and caprine ETX intoxication. Microscopically, a microangiopathy, with deposition of a protein-rich perivascular fluid, is found in most acute ovine case, but in less than 20% of caprine cases. Cerebellar coning, due to an uncompensated increase in ICP, is also regarded as highly diagnostically suggestive but not confirmatory of type D enterotoxemia. In ovine but not caprine cases following a more protracted clinical course, bilaterally symmetrical necrotic foci are sometimes found in certain selectively vulnerable neuroanatomic regions, a pattern not observed with the same distribution in any other ruminant neurologic disorder.

While these neuropathologic changes are generally sufficient for the diagnostic pathologist to make an etiological diagnosis, much remains to be elucidated about the different events marking the journey of ETX from the intestinal lumen through the systemic circulation to the brain. It is also unclear why certain brain regions, and/or their microvasculature, appear to be selectively vulnerable to ETX-induced injury. Pathogenetic studies are important; although vaccination has reduced the occurrence of this disease in ruminants, clinical cases still occur too commonly, with concomitant economic loss. Moreover, ETX is a potential zoonosis and bioterrorism threat. Since a wide range of species are known to be susceptible to this potent neurotoxin, it is likely that humans are also at risk, particularly as ETX is the third most potent clostridial toxin after botulism and tetanus.

ETX was classified by the Centers for Disease Control and Prevention in the United States as a select agent—that is, one determined to pose a potential threat to public health and safety—but it has now been removed from this list. However, ETX is still classified as a potential biological weapon in some countries and authorization is required for its laboratory use. There is currently no therapy or vaccine approved for use in humans if ETX were to be used in a nefarious manner [87].

## Figures and Tables

**Table 1:** Toxinotyping of *Clostridium perfringens* (modified from Rood et al., 2018).

Type	Typing Toxins					
	Alpha (CPA)	Beta (CPB)	Epsilon (ETX)	Iota (ITX)	Enterotoxin (CPE)	Necrotic Enteritis B-like Net-B
A	+	−	−	−	−	−
B	+	+	+	−	−/+	−
C	+	+	−	−	−/+	−
D	+	−	+	−	−/+	−
E	+	−	−	+	−/+	−
F	+	−	−	−	+	−
G	+	−	−	−	−/+	+

## Data Availability

The contents of this review are available on request from the corresponding author (F.A.U.).
